# Supplementary material for: Analysis of the antimicrobial activity of zinc oxide nanoparticles against drug-resistant bacteria and their applications in the disinfection process
Source: PLoS One. 2026 Feb 13;21(2):e0340470. doi: 10.1371/journal.pone.0340470 (PMC12904420; doi:10.1371/journal.pone.0340470)
Supplement: S1 Table — (DOCX) [file pone.0340470.s001.docx]

| **Table S1:** The mean of MIC values of the tested microorganisms. | | | | | | |
| --- | --- | --- | --- | --- | --- | --- |
| **No.** | **Microorganisms** | **MIC values / relative ZnO-NPs concentration (µg/mL)** | | | **The mean of the MIC values (MIC_50_)*** | **Relative ZnO-NPs Conc. (µg/mL) at MIC_50_** |
|  |  | **Experiment 1** | **Experiment 2** | **Experiment 3** |  |  |
| 1 | ***ESBL +ve*** | 256/39 | 256/39 | 512/19 | 256 | 39 |
| 2 | ***ESBL -ve*** | 512/19 | 512/19 | 512/19 | 512 | 19 |
| 3 | ***A.baumannii MDR*** | 64/ 150 | 64/150 | 64/150 | 64 | 150 |
| 4 | ***A.baumannii MS*** | 64/150 | 128/70 | 128/70 | 128 | 70 |
| 5 | ***P.aeruginosa CRPA*** | 32/310 | 32/310 | 64/150 | 32 | 310 |
| 6 | ***P.aeruginosa CSPA*** | 64/150 | 64/150 | 64/150 | 64 | 150 |
| 7 | ***S.aureus MRSA*** | 512/19 | 512/19 | 512/19 | 512 | 19 |
| 8 | ***S.aureus MSSA*** | 256/39 | 256/39 | 256/39 | 256 | 39 |
| 9 | ***E.faecium VRE*** | 256/39 | 256/39 | 256/39 | 256 | 39 |
| 10 | ***E.faecium VSE*** | 1024/9.7 | 1024/9.7 | 1024/9.7 | 1024 | 9.7 |
| 11 | ***S.pneumoniae*** | 1024/9.7 | 1024/9.7 | 1024/9.7 | 1024 | 9.7 |
| 12 | ***Vibrio cholera*** | 512/19 | 512/19 | 512/19 | 512 | 19 |
| 13 | ***Aspergillus spp.*** | 64/150 | 64/150 | 64/150 | 64 | 150 |
| 14 | ***Candida glabrata*** | 256/39 | 512/19 | 512/19 | 512 | 19 |
| 15 | ***Candida albicans*** | 64/150 | 64/150 | 64/150 | 64 | 150 |
| 16 | ***Candida parapsilosis*** | 128/70 | 128/70 | 128/70 | 128 | 70 |
| 17 | ***H.influenzae*** | 64/150 | 64/150 | 64/150 | 64 | 150 |
| 18 | ***Brucella sp.*** | 1024/9.7 | 1024/9.7 | 1024/9.7 | 1024 | 9.7 |
|  | | | | | | |

* The mean of MIC represents the average value across the dilution series and calculated by arranging the MIC values in ascending order then the middle value represent the concentration that inhibit 50% of isolates (also termed MIC_50_ values). Abbreviations: Minimal inhibitory concentration, MIC; Zinc oxide- Nanoparticles, ZnO-NPs; Extended spectrum beta-lactamase, ESBL; Multi-drug resistance, MDR; Multi-drug susceptible, MS; Carbapenem-resistance *Pseudomonas aeruginosa*, CRPA; Carbapenem-susceptible *Pseudomonas aeruginosa* CSPA; Methicillin- resistant *Staphylococcus aureus*, MRSA; Methicillin- susceptible *Staphylococcus aureus,* MSSA; Vancomycin resistant *Enterococcus faecium* VRE; Vancomycin sensitive *Enterococcus faecium* VSE
