# Supplementary material for: Analysis of the antimicrobial activity of zinc oxide nanoparticles against drug-resistant bacteria and their applications in the disinfection process
Source: PLoS One. 2026 Feb 13;21(2):e0340470. doi: 10.1371/journal.pone.0340470 (PMC12904420; doi:10.1371/journal.pone.0340470)
Supplement: S2 Table — (DOCX) [file pone.0340470.s002.docx]

|  |  | | | | | | | | |  |  |  |
| --- | --- | --- | --- | --- | --- | --- | --- | --- | --- | --- | --- | --- |
| **Disc contents** | **Table S2 The mean of inhibition zone diameter for Gram negative bacteria using disc diffusion assay** | | | | | | | | | | | |
|  | ***ESBL positive*** | | ***ESBL negative*** | | ***A.baumannii MDR*** | | ***A.baumannii MS*** | | ***CPRA*** | | ***P. aeruginosa*** | |
|  | ***Zone mm*** | ***I**** | ***Zone mm*** | ***I*** | ***Zone mm*** | ***I*** | ***Zone mm*** | ***I*** | ***Zone mm*** | ***I*** | ***Zone mm*** | ***I*** |
| **ZnO-NPs** | 19.4 |  | 19.6 |  | 16 |  | 16.7 |  | 17.1 |  | 13 |  |
|  | 19.3/19.4/19.5/19.4 |  | 19.4/19.6/19.7/19.7 |  | 15.8/16.0/16.1/16.1 |  | 16.6/16.7/16.7/16.8 |  | 17.0/17.1/17.1/17.2 |  | 12.9/13.0/13.1/13.0 |  |
| **ZnA** | No zone |  | 9 |  | No zone |  | 9 |  | No zone |  | No zone |  |
|  | No zone |  | 8.9/9.0/9.1/9.0 |  | No zone |  | 8.9/9.0/9.1/9.0 |  | No zone |  | No zone |  |
| **Amp** | No zone | R | No zone | R | No zone | R | 9 | R | No zone | R | No zone | R |
|  | No zone |  | No zone |  | No zone |  | 8.9/9.0/9.1/9.0 |  | No zone |  | No zone |  |
| **CAZ** | 16 | R | 21 | S | No zone | R | 19 | I | 16.8 | I | 24 | S |
|  | 15.9/16.0/16.1/16.0 |  | 20.8/20.9/21.1/21.2 |  | No zone |  | 18.8/18.9/19.1/19.2 |  | 16.7/16.8/16.8/16.9 |  | 23.8/23.9/24.1/24.2 |  |
| **ETP** | 23 | S | 27.6 | S | No zone | R | 11 | R | No zone | R | 9 | R |
|  | 22.8/23.0/23.1/23.1 |  | 27.4/27.5/27.7/27.8 |  | No zone |  | 10.8/10.9/11.1/11.2 |  | No zone |  | 8.9/9.0/9.1/9.0 |  |
| **CN** | 13.4 | I | 16.7 | S | 11.6 | R | 20 | S | 19 | S | 16.8 | S |
|  | 13.3/13.4/13.5/13.4 |  | 16.5/16.6/16.8/16.9 |  | 11.4/11.5/11.7/11.8 |  | 19.8/19.9/20.1/20.2 |  | 18.8/18.9/19.1/19.2 |  | 16.7/16.8/16.8/16.9 |  |

The table shows raw data from four experimental repeats. The average (mean) zone diameters are highlighted in blue boxes, with the individual zone diameter measurements from each experiment listed below the respective mean. * Presents the interpretation (I) of zone diameters, categorizing them as Susceptible (S), Intermediate (I), or Resistant (R) based on the antibiogram breakpoints. Abbreviations: Zinc oxide-nanoparticles, ZnO-NPs; Zinc acetate, ZnA; Extended-spectrum beta-lactamases, ESBLs; Multidrug-resistant bacteria, MDR; Carbapenem-resistant *Pseudomonas aeruginosa*, CRPA; *A. baumannii* Multisensitive, *A. baumannii* MS; Ampicillin, Amp; Ceftazidime, CAZ; Ertapenem, ETP; Gentamicin, CN.

.
