# Supplementary material for: Analysis of the antimicrobial activity of zinc oxide nanoparticles against drug-resistant bacteria and their applications in the disinfection process
Source: PLoS One. 2026 Feb 13;21(2):e0340470. doi: 10.1371/journal.pone.0340470 (PMC12904420; doi:10.1371/journal.pone.0340470)
Supplement: S3 Table — (DOCX) [file pone.0340470.s003.docx]

| **Discs Contant** | **Table S3. The mean of inhibition zone diameter for Gram positive bacteria using disc diffusion assay** | | | | | | | | |  |
| --- | --- | --- | --- | --- | --- | --- | --- | --- | --- | --- |
|  | ***MRSA*** | | ***MSSA*** | | ***VRE*** | | ***VSE*** | | ***S.pneumoniae*** | |
|  | ***Zone mm*** | ***I**** | ***Zone mm*** | ***I*** | ***Zone mm*** | ***I*** | ***Zone mm*** | ***I*** | ***Zone mm*** | ***I*** |
| **ZnO-NPs** | 24 |  | 17 |  | 13.4 |  | 14.1 |  | 23 |  |
|  | 23.8/23.9/24.1/24.2 |  | 16.7/16.9/17.1/17.3 |  | 13.2/13.3/13.5/13.6 |  | 13.9/14.0/14.2/14.3 |  | 22.8/22.9/23.1/23.2 |  |
| **ZnA** | 11.9 |  | 10.4 |  | No zone |  | No zone |  | 12.4 |  |
|  | 11.8/11.9/11.9/12.0 |  | 10.3/10.4/10.5/10.4 |  | No zone |  | No zone |  | 12.3/12.4/12.5/12.4 |  |
| **P** | No zone | R | 14.7 | R | No zone | R | No zone | R | 26 | S |
|  | No zone |  | 14.6/14.7/14.8/14.7 |  | No zone |  | No zone |  | 25.8/26.1/26.2/25.9 |  |
| **OX** | No zone | R | 20 | S | No zone | R | No zone | R | No zone | R |
|  | No zone |  | 19.7/20.1/20.2/19.9 |  | No zone |  | No zone |  | No zone |  |
| **VA** | 19.7 | S | 17.3 | S | No zone | R | 23.4 | S | 24 | S |
|  | 19.5/19.6/19.8/20.0 |  | 17.1/17.2/17.4/17.5 |  | No zone |  | 23.1/23.3/23.5/23.7 |  | 23.8/23.9/24.1/24.2 |  |
| **LZD** | 35 | S | 28 | S | 24.7 | S | 27.5 | S | 30 | S |
|  | 34.7/34.9/35.1/35.3 |  | 27.7/27.9/28.1/28.3 |  | 24.5/24.6/24.8/24.9 |  | 27.3/27.4/27.6/27.7 |  | 29.5/30.0/30.5/30.0 |  |
|  |  |  |  |  |  |  |  |  |  |  |

The table shows raw data from four experimental repeats. The average (mean) zone diameters are highlighted in blue boxes, with the individual zone diameter measurements from each experiment listed below the respective mean. * Presents the interpretation (I) of zone diameters, categorizing them as Susceptible (S), Intermediate (I), or Resistant (R) based on the antibiogram breakpoints. Abbreviations: Zinc oxide-nanoparticles, ZnO-NPs; Zinc acetate, ZnA; Methicillin-resistant *Staphylococcus aureus*, MRSA; Methicillin-susceptible *Staphylococcus aureus*, MSSA; Vancomycin-susceptible *Enterococcus*, VSE; Penicillin, P; Oxacillin, OX; Vancomycin, VA; Linezolid, LZD.
