# Supplementary material for: Analysis of the antimicrobial activity of zinc oxide nanoparticles against drug-resistant bacteria and their applications in the disinfection process
Source: PLoS One. 2026 Feb 13;21(2):e0340470. doi: 10.1371/journal.pone.0340470 (PMC12904420; doi:10.1371/journal.pone.0340470)
Supplement: Supplementary file 4 [file pone.0340470.s004.docx]

Table S4: Percent of reduction in CFU/mL calculations on each sample type after exposure to ZnO-NPs at different time intervals performed by pour plate assay

Time of exposure	Water				Glass				Stainless steel			
	Exp.1 CFU/mL	Exp.2 CFU/mL	*CFU/mL	% Of reduction	Exp.1 CFU/mL	Exp.2 CFU/mL	*CFU/mL	% Of reduction	Exp.1 CFU/mL	Exp.2 CFU/mL	*CFU/mL	% Of reduction
0 min	2590	2710	2650	-	927	973	950	-	500	540	520	-
5 mins	2130	2210	2170	4.8	635	825	730	2.2	432	468	450	0.7
10 mins	1547	1693	1620	10.3	500	540	520	4.3	320	300	310	2.1
15 mins	953	867	910	17.4	363	277	320	6.3	233	227	230	2.9
20 mins	810	790	800	18.5	215	305	260	6.9	163	177	170	3.5
25 mins	690	650	670	19.8	213	147	180	7.7	100	120	110	4.1

* CFU/ml represent the mean of CFU/ml of the duplicate experimental repeats. Abbreviations: Colony forming units per millilitre, CFU/mL.
