# Supplementary material for: Analysis of the antimicrobial activity of zinc oxide nanoparticles against drug-resistant bacteria and their applications in the disinfection process
Source: PLoS One. 2026 Feb 13;21(2):e0340470. doi: 10.1371/journal.pone.0340470 (PMC12904420; doi:10.1371/journal.pone.0340470)
Supplement: Attachment — Submitted filename: Response to Reviewers.pdf [file pone.0340470.s007.pdf]

Response to reviewers

We sincerely thank the reviewer for the careful and constructive evaluation of our manuscript. We greatly appreciate the time and effort devoted to providing detailed comments and suggestions, which have helped us improve the clarity, accuracy, and overall quality of our work. We have carefully addressed each point raised, and the manuscript has been revised accordingly to reflect these improvements.

In addition, we have thoroughly revised the manuscript to improve language and clarity. All data presented in the tables are now also included in the supplementary materials, with the addition of two new supplementary files. Furthermore, the figures in the manuscript provide visual confirmation of the antimicrobial effects, as assessed using multiple complementary methodologies.

Reviewer #1: Your manuscript addresses an important area of research, particularly in the context of antimicrobial resistance and the potential of nanomaterials in disinfection. The work is promising; however, several clarifications and corrections are needed to improve transparency, reproducibility, and clarity. Please consider the following comments:

Response to Reviewer 1 comments:

Comment 1: Fungal assay controls: You mention the use of E. coli ATCC 25922, S. aureus ATCC 25923, and P. aeruginosa ATCC 27853 as standard quality control strains for antibacterial assays. However, it is not clear what reference strains or controls were used for the antifungal assays. Please provide this information.

Response to comment 1: Thank you for your valuable comment and for highlighting the need for clarification regarding the fungal reference strains used in the antifungal assays. We confirm that standard fungal isolates were used in our study, as listed in Table 1. In accordance with your valuable suggestion, we have revised the relevant paragraph in the Methods section to include the ATCC numbers of all standard fungal isolates employed: *Candida albicans* ATCC 90028, *Candida parapsilosis* ATCC 22019, and *Candida glabrata* ATCC 2001. Additionally, we have included *Haemophilus influenzae* ATCC 49247 to ensure that the description fully reflects all ATCC strains utilized in this work. These details have now been clearly incorporated into the manuscript to improve transparency and methodological clarity.

Comment 2: Plant material collection:
If plants were used in nanoparticle synthesis, the site of collection must be mentioned, along with details of botanical authentication (voucher specimen, herbarium record, or expert identification). This is important for reproducibility.

Response to comment 2: Thank you for your thoughtful comment. We appreciate the importance of providing complete information on plant material collection to ensure reproducibility. In this study, the plant material used for nanoparticle synthesis consisted of root hairs from *Phoenix dactylifera*. These plants were cultivated and collected from the campus of Al-Ahliyya Amman University (Amman, Jordan), ensuring a controlled and well-documented source.
The botanical identification of *Phoenix dactylifera* was verified by an expert botanist in the Faculty of Agricultural Technology at Al-Ahliyya Amman University, based on established morphological criteria. Although a voucher specimen was not prepared, the identification was

conducted by qualified personnel, and this information has now been clearly incorporated into the revised manuscript to ensure full transparency and traceability of the plant material used.
We believe that this additional clarification fully addresses the reviewer's concern and strengthens the methodological rigor of our study.

Comment 3: Equipment, reagents, and chemicals
Provide the make, model, and country of origin for the instruments (e.g., spectrophotometer, centrifuge, electron microscope) and for all key reagents and chemicals used in the experiments.

Response to comment 3: Thank you for your helpful comment. We agree that providing complete information on the equipment, reagents, and chemicals used in this study is essential for reproducibility and methodological transparency. In response, we have revised the Materials and Methods section to include the make, model, and country of origin for all major instruments utilized.
Additionally, we have provided full details for all key reagents and chemicals employed in the experiments, including the manufacturer and country of origin. These additions ensure that the experimental procedures can be accurately replicated and that all methodological specifications are fully traceable.

Comment 4: Correction in terminology:
The statement "The antibacterial activity of synthesized ZnO-NPs was assessed against fungi" is incorrect and should be revised to "The antifungal activity of synthesized ZnO-NPs was assessed against fungi."

Response to comment 4: Thank you for bringing this terminology issue to our attention. We fully agree with your observation. The statement has been corrected from "The antibacterial activity of synthesized ZnO-NPs was assessed against fungi" to "The antifungal activity of synthesized ZnO-NPs was assessed against fungi."

Comment 5: Clarification of ZnO_2-NPs:
The manuscript refers to ZnO_2-NPs. Please clarify whether this is a typographical error for ZnO-NPs, or if zinc peroxide nanoparticles (ZnO_2) were also synthesized and tested. Consistency in chemical nomenclature is critical.

Response to comment 5: Thank you for your careful reading and for pointing out the inconsistency in the nanoparticle nomenclature. We apologize for the typographical errors in the manuscript. To clarify, only zinc oxide nanoparticles (ZnO-NPs) were synthesized and tested in this study. The term "ZnO_2-NPs" appeared mistakenly in two places and does not reflect the materials used. We have carefully reviewed the entire manuscript and corrected these occurrences to ensure full consistency and accuracy in chemical nomenclature. We appreciate your attention to detail, as this correction helps improve the clarity and scientific precision of our work.
