# Supplementary material for: Analysis of the antimicrobial activity of zinc oxide nanoparticles against drug-resistant bacteria and their applications in the disinfection process
Source: PLoS One. 2026 Feb 13;21(2):e0340470. doi: 10.1371/journal.pone.0340470 (PMC12904420; doi:10.1371/journal.pone.0340470)
Supplement: Attachment — Submitted filename: Response to reviewer.docx [file pone.0340470.s005.docx]

**Response to reviewer**

**Editor Comments:**

**Comment:** Aim of the work not clear.

**Response:** Thank you for your valuable feedback. We have addressed this comment by clearly stating the specific aim of the study at the end of the Introduction section to enhance clarity and focus.

**Comment:** The novelty should be described more clearly.

**Response:** Thank you for your insightful comment. We have revised the Introduction to explicitly highlight the novelty of our study, emphasizing the unique use of Phoenix dactylifera root extract and the comprehensive antimicrobial evaluation of ZnO-NPs on clinical isolates and environmental surfaces.

**Comment:** what about the chemical composition of extract and what about the characterization of  Zinc Oxide Nanoparticles.

**Response:** Thank you for your observation. We want to clarify that this study builds upon our previously published work by Naser et al. (2021), which provided a detailed account of the chemical composition of the Phoenix dactylifera extract and a comprehensive physicochemical characterization of the synthesized ZnO nanoparticles. To avoid redundancy, the current manuscript evaluates the biological efficacy of these well-characterized nanoparticles against multidrug-resistant pathogens. A clarification has now been added to the revised manuscript's Zinc Oxide Nanoparticles Synthesis section.

**Comment:** Figures   must be improved( resolution improved).

**Response:** Thank you for your valuable comment. We appreciate your suggestion regarding the quality of the figures. In response, we have carefully revised all the figures in the manuscript and improved their resolution to ensure better clarity and visual representation of the data.

**Comment:** References must be revised

**Response:** Thank you for your comment. The references have been thoroughly revised and formatted according to the NLM (National Library of Medicine) style guidelines.

**Comment:** Please, check typing errors and punctuations and correct errors in the revised version.

**Response:** Thank you for your suggestion. We have carefully proofread the entire revised manuscript and corrected all typing, punctuation, and grammatical errors.

**Reviewer 1**

**Comment:** At some places, typos and repetition of words are present.

**Response:** Thank you for your valuable observation regarding the presence of typographical errors and repetition in the manuscript. We have thoroughly revised the entire manuscript, carefully correcting all identified typographical mistakes and removing repetitive words to improve the clarity, accuracy, and overall quality of the text.

**Comment:** The study presented needs to highlight the research gaps and novelty of this study.

**Response:** Thank you for your valuable comment. We have revised the introduction to clearly highlight the research gap and the novelty of our study by emphasizing the underexplored use of Phoenix dactylifera root extract for green synthesis of ZnO-NPs and their unique application in disinfecting clinically relevant MDR isolates, surfaces, and water an area not widely reported in previous literature.

**Comment:** The ZnO nanoparticles were extracted from the roots of Phoenix dactylifera: seems incorrect statement. Redraft

**Response:** Thank you for your insightful comment. We have revised the sentence to clarify that the roots of Phoenix dactylifera were used to prepare the plant extract for the green synthesis of ZnO nanoparticles, ensuring technical accuracy.

**Comment:** Which was the quality control strain used in the antibacterial assays?

**Response:** Thank you for your valuable comment. We have addressed this by explicitly stating in the revised methodology that E. coli ATCC 25922, S. aureus ATCC 25923, and P. aeruginosa ATCC 27853 were used as standard quality control strains in the antibacterial assays.

**Comment:** Why was the disc diffusion assay performed in addition to the MIC assays?]

**Response:** Thank you for your valuable comment. We have addressed your concern by clarifying that the disc diffusion assay was performed alongside MIC to confirm antimicrobial activity visually and to compare inhibition zones with standard antibiotic responses.

**Comment:** 'The Diluent control bottle was also supplemented with 1 ml of 0.1317 g/ml Zinc acetate' what was the purpose of this?

**Response:** Thank you for your insightful comment. We have clarified in the revised manuscript that the diluent control containing zinc acetate was included to confirm that the antimicrobial activity observed was explicitly due to ZnO-NPs and not the precursor compound alone.

**Comment:** Explain the green synthesis employed for the ZnO NPs, so far? What is the highlight of this study?

**Response:** Thank you for your valuable comment. We have clarified that a green synthesis method utilizing Phoenix dactylifera root extract was employed. This study's unique contribution lies in its comprehensive evaluation of biosynthesized ZnO-NPs against multidrug-resistant pathogens and their practical use in surface and water disinfection.

**Comment:** Quality of figures appear too poor to be assessed.

**Response:** Thank you for your valuable comment regarding the quality of the figures. We have carefully addressed this comment by improving the resolution, clarity, and overall presentation of all figures in the manuscript. Specifically, Figure 1 has been completely reformatted to provide a clearer understanding of the MIC setup performed in the study. Figure 2 has been replaced with a higher-resolution image to enhance its clarity. For Figure 4, we have not only improved the image quality but also added additional images to cover all the tested microorganisms for a more comprehensive representation. Figure 5 has been enhanced by increasing the size and improving its clarity for better visibility. Additionally, Figure 6 has been removed as it was a repetitive representation of the same data already presented in Table 5. The previous Figure 7 (now Figure 6) has been modified by increasing its size and improving clarity, and the previous Figure 8 (now Figure 7) has also been revised for better size and visibility. Furthermore, all figure legends throughout the manuscript have been rechecked and revised for language, grammar, and clarity, ensuring accurate and concise descriptions that support the improved figures. We believe these changes have significantly enhanced the overall quality and readability of the figures in the revised manuscript.

**Reviewer 2**

**Comment:** “dried in a 40 °C oven” should be “dried in an oven at 40 °C ”

**Response:** Thank you for your observation. We have revised the phrase to “dried in an oven at 40 °C” by your suggestion.

**Comment:**  Gram should be g.

**Response:** Thank you for your observation. We have corrected all instances where "Gram" was incorrectly used as a unit and replaced it with the standard symbol "g" throughout the manuscript.

**Comment:** ml should be mL.

**Response:** Thank you for your valuable feedback. We have revised the manuscript to ensure that all instances of "ml" have been corrected to the standard unit format "mL" by scientific conventions.

**Comment:** Provide formula for zinc acetate

**Response:** Thank you for your comment; we have added the chemical formula of zinc acetate $(Zn\left( CH_{3}COO \right)_{2}.2H_{2}O)$ in the synthesis section as suggested.

**Comment:** Correct grammar in yield formula.

**Response:** Thank you for your observation. The revised manuscript has been corrected for grammatical and clarity issues in the yield formula.

**Comment:** Please update your introduction by citing these recent references of greenly synthesized nps

**Response:** Thank you for your valuable suggestion. We have thoroughly revised the introduction section by incorporating all the recent references related to greenly synthesized ZnO nanoparticles provided by the reviewer, ensuring improved contextual relevance and scientific depth.

**Comment:** The manuscript lacks the characterization of synthesized ZnO Nps. Provide UV-Vis, XRD, FT-IR, SEM analyses

**Response:** Thank you for your valuable comment. The comprehensive physicochemical characterization of the synthesized ZnO nanoparticles—including UV–Vis, FTIR, XRD, and particle size analysis—was previously performed and published in our earlier work (Naser et al., 2021). As this study is a continuation of a research series utilizing the same batch of well-characterized nanoparticles, we have focused here on their antimicrobial efficacy. Repeating the characterization would be redundant; however, the relevant data can be accessed in the cited references. We acknowledge the importance of including zeta potential analysis to assess nanoparticle surface charge and colloidal stability, and we are addressing this to ensure full compliance with the reviewer’s feedback.

References

Naser, R., Abu-Huwaij, R., Al-khateeb, I., Abbas, M. M., & Atoom, A. M. (2021). Green synthesis of zinc oxide nanoparticles using the root hair extract of Phoenix dactylifera: Antimicrobial and anticancer activity. *Applied Nanoscience*, *11*, 1747-1757.

Abu-Huwaij, R., Abbas, M. M., Al-Shalabi, R., & Almasri, F. N. (2022). Synthesis of transdermal patches loaded with greenly synthesized zinc oxide nanoparticles and their cytotoxic activity against triple negative breast cancer. *Applied Nanoscience*, *12*, 69-78.

Obaid, R. Z., Abu-Huwaij, R., & Hamed, R. (2023). Development and Characterization of Anticancer Model Drug Conjugated to Biosynthesized Zinc Oxide Nanoparticles Loaded into Different Topical Skin Formulations. *Jordan Journal of Pharmaceutical Sciences*, *16*(2), 486-486.

**Comment:** Antimicrobial properties are highly size and stability dependent. Calculate size of the NPs. Use zeta-potential to determine charge and stability of the NPs.

**Response:** Thank you for your valuable comment. In response, we have included the size and zeta potential analysis of the synthesized ZnO-NPs. The nanoparticles showed an average size of 22.57 ± 4.79 nm with a PDI of 0.53 ± 0.02, and a zeta potential of −19.23 ± 1.40 mV, indicating moderate uniformity and good colloidal stability key factors supporting their antimicrobial efficacy.

**Comment:** Provide overlaid FTIR of all entities involved. Highlight the areas of any change.

**Response:** Thank you for your valuable comment. This manuscript is part of an ongoing research series of Prof. Rana Abu-Huwaij, with this being the sixth publication on zinc oxide nanoparticles. The comprehensive characterization of the synthesized ZnO NPs, including UV–Vis, XRD, and FTIR analyses, was thoroughly conducted and documented in earlier studies. Specifically, FTIR analyses of all relevant entities including the Phoenix dactylifera extract, zinc acetate precursor, and ZnO-NPs were presented with overlaid spectra in previous publications. These spectra highlighted key spectral shifts confirming the involvement of functional groups in nanoparticle formation and stabilization. As the current manuscript focuses on antimicrobial evaluation and utilizes the same well-characterized ZnO NPs, repeating these analyses was deemed redundant. Readers are referred to the following published references (35, 38-41) for detailed FTIR profiles and spectral interpretations.

**Comment:** Provide a table of FT-IR assignments showing wavenumber against each functional group.

**Response:** Thank you for your comment. Our earlier publications thoroughly presented a detailed FT-IR analysis, including wavenumber assignments for all functional groups, using the same ZnO-NPs batch. Therefore, we have referenced those studies (35, 38-41) for complete spectral and tabulated data to avoid redundancy.

**Comment:** The study did not provide details on the interaction mechanism between NPs and bacterial strains, limiting our understanding of how the NPs function.

**Response:** Thank you for your valuable comment. To clarify their antimicrobial action, we have now included a detailed explanation in the Discussion section describing the known interaction mechanisms of ZnO-NPs with bacterial strains, including ROS generation, Zn²⁺ ion release, and membrane disruption.

**Comment:** The study tested only certain concentrations for the minimum inhibitory concentration (MIC), leaving it unclear whether the concentration range should be extended to identify the optimal concentration for different bacterial strains.

**Response:** Thank you for your insightful comment. We have clarified the range of concentrations tested for MIC (from 5 × 10³ to 2.4 µg/mL) and noted that this range was selected to ensure broad coverage across bacterial susceptibilities. The revised section now explicitly mentions this range and its rationale.

**Comment:** The use of the disk diffusion method may not fully capture antibacterial activity under real-world conditions; therefore, additional experimental methods are required to validate the results.

**Response:** Thank you for your insightful comment. We agree that the disk diffusion method has limitations in replicating real-world conditions. To address this, we have supplemented it with broth microdilution MIC assays, pour plate disinfection tests, and VersaTrek Mycobottle evaluations to comprehensively validate the antibacterial efficacy of ZnO-NPs.

**Comment:** Would alternative testing methods, such as liquid bacterial culture, provide a more accurate evaluation of antibacterial activity?

**Response:** Thank you for your insightful suggestion. We want to clarify that the MIC testing in our study was conducted using the broth microdilution method, a liquid-based bacterial culture assay. This method is widely accepted as a standard and provides a more accurate and quantitative evaluation of antibacterial activity compared to solid-based approaches such as the disc diffusion method.

**Comment:** Is it necessary to broaden the concentration range tested for MIC to determine the optimal concentration for various bacterial strains?

**Response:** Thank you for your insightful comment. We agree that broadening the concentration range could help determine more strain-specific MIC values. While our selected range was based on prior literature and preliminary screening, we have now acknowledged this limitation in the revised manuscript's Discussion section and suggested that future studies consider extended or finer concentration gradients for optimized evaluation.

**Comment:** Can NPs be incorporated into real-life antibacterial products such as hand sanitizers and disinfectants, or used in other medical applications?

**Response:** Thank you for your valuable suggestion. We have addressed the potential real-life applications of ZnO-NPs, including their incorporation into hand sanitizers, disinfectants, wound dressings, and medical device coatings, in the Discussion section. We have also emphasized the need for further studies on formulation, stability, and safety to support their clinical or commercial use.

**Comment:** Compare antibacterial activity using following greenly synthesized NPs

**Response:** Thank you for your valuable suggestion. A comparative analysis has been incorporated into the Discussion section, highlighting the antibacterial efficacy of our green-synthesized ZnO-NPs relative to other recent AgNP-based systems (e.g., Jabbar et al., Siddique et al., Ullah et al.). Our ZnO-NPs exhibited broader activity against both Gram-positive and Gram-negative bacteria, with inhibition zones reaching up to 24 mm, and demonstrated effective surface and water disinfection. These findings confirm their strong antimicrobial potential.

**Comment:** There are numerous mistakes in terms of grammar and typos.

**Response:** Thank you for pointing out the grammatical and typographical errors. We have carefully revised the entire manuscript to correct errors in grammar, punctuation, and wording, thereby enhancing clarity and readability throughout the text.

**Comment:** Please report results in the conclusion section. What are the future aspects of the research work conducted?

**Response:** Thank you for your insightful comment. We have revised the Conclusion section to include key antimicrobial results, such as MIC values, inhibition zones, and surface disinfection efficacy. We have also outlined future directions, including formulation development, synergy with antibiotics, clinical applications, and assessments of biocompatibility and scalability.

**Reviewer #3**

**Comment:** In zinc nanopartical sysnthesis in formula acetate spelling correction needed

**Response:** Thank you for your observation. The spelling of “zinc acetate” has been thoroughly reviewed and is now correctly addressed throughout the manuscript, particularly in the section titled “Zinc oxide nanoparticles synthesis.” We appreciate your attention to detail.

**Comment:** Author has to prove that the nano particals are formed by doing various analytical methods, then only other thigs u can prove, line antibactierial

**Response:** Thank you for your feedback. The ZnO nanoparticles (NPs) used in this study were previously synthesized and fully characterized (Naser et al., 2021) using UV–Vis, FTIR, XRD, and particle size analysis, confirming the formation and composition of the nanoparticles. This has been referenced in the “Zinc oxide nanoparticles synthesis” section, as the current work focuses on their biological efficacy.

**Comment:** In table 4 first coumn description is missing like ( Amp, CAZ atc)

**Response:** Thank you for your valuable observation. As suggested, we have revised Table 4 to include full descriptions of all antimicrobial discs used (e.g., Penicillin, Oxacillin, Vancomycin, etc.) in the first column. The antibiotic abbreviations have also been clarified in the table footnote to ensure clarity for readers.

**Comment:**  Drug resistance bacteria is not reflected in title

**Response:** Thank you for your valuable feedback. We agree that the title should reflect the study's focus on drug-resistant pathogens. Accordingly, we have revised the title to “Analysis of the Antimicrobial Activity of Zinc Oxide Nanoparticles Against Drug-Resistant Bacteria and Their Applications in the Disinfection Process” This change ensures the title accurately represents the content and objectives of the study.

**Comment:** compare the animicrobial results with plant extract also find the comparision study

**Response:** Thank you for your insightful comment. We have addressed this in the Results and Discussion sections by comparing the antimicrobial effects of the crude Phoenix dactylifera extract with those of the synthesized ZnO-NPs. The plant extract exhibited minimal inhibition, with zones of inhibition of 7.5 mm for E. coli and 9.2 mm for MRSA. In comparison, ZnO-NPs demonstrated significantly larger zones (19.6 mm and 24.0 mm, respectively), confirming that the enhanced antimicrobial activity is due to nanoparticle synthesis rather than the extract alone.

**Reviewer #4**

**Comment:** The typographical errors should be addressed immediately. Terms like in vitro and via should be in italics

**Response:** Thank you for pointing out the typographical formatting issues. We have carefully reviewed the manuscript and revised all relevant Latin terms, including in vitro and via, to appear in italics as per scientific writing conventions. These corrections have been applied consistently throughout the text.

**Comment:** At few places Phoenix dactylifera is not in italics. Kindly correct it.

**Response:** Thank you for your observation. We have thoroughly reviewed the manuscript and corrected all instances of Phoenix dactylifera to ensure it is consistently italicized in accordance with botanical nomenclature guidelines.

**Comment:** Terms like ml, µl should be written as mL, µL.

**Response:** Thank you for pointing this out. We have reviewed the manuscript and corrected the units to follow standard SI conventions. All instances of "ml" and "µl" have been updated to "mL" and "µL" respectively.

**Comment:** Fig 3, 4 ,5 and 8 are hazy. The authors must give original clear images

**Response:** Thank you for your constructive comment regarding the clarity of Figures 3, 4, 5, and 8. We have carefully addressed this concern and replaced all the mentioned figures with original high-resolution images to ensure better clarity and visibility. Specifically, Figures 3, 4, 5, and 8 have been updated with clearer images that accurately represent the experimental results. Additionally, the size and clarity of Figure 5, the revised Figure 6 (previously Figure 7), and Figure 7 (previously Figure 8) have also been improved to enhance visual presentation. Furthermore, Figure 6 has been removed from the manuscript as the data it presented was repetitive and already well-represented in Table 5.

**Reviewer #5**

**Comment:** The manuscript, Analysis of the Antimicrobial Activity of Zinc Oxide Nanoparticles and Their Applications in Disinfection Process, has been written well but it needs major revisions, There is need to show the results of SEM (Scanning Electron Microscope) photograph to show the size of nano particle as there is no any information available about the nano particle.

**Response:** Thank you for your valuable observation. The particle size of the ZnO nanoparticles was determined using a Nano Zetasizer (Zen 3600, Malvern, U.K.), revealing an average size of 22.57 ± 4.79 nm with a PDI of 0.53 ± 0.02. Additionally, the zeta potential was measured at −19.23 ± 1.40 mV, indicating good colloidal stability. While SEM analysis was previously presented in our published studies using the same batch of nanoparticles, we have now revised and enhanced the figures illustrating inhibition zones in the current manuscript for improved clarity.

**Comment:** There is need to show the results of SEM (Scanning Electron Microscope) photograph to show the size of nano particle as there is no any information available about the nano particle.

**Response:** Thank you for your comment. The ZnO nanoparticles used in this study, including SEM imaging, were previously characterised, as reported in our earlier publication (Naser et al., 2021). The nanoparticles had a size range of 30.87–47.89 nm, with an average size of 36.23 nm and a PDI of 0.45 (Nano Zetasizer, Malvern, UK). As the same batch was used here, we have referenced the original SEM data in the Methods section rather than repeating it.

**Comment:** The figures about the inhibition zones are not clear.

**Response:** Thank you for your valuable comment regarding the clarity of the figures representing the inhibition zones. We have carefully addressed this concern and replaced the respective figures with original high-resolution images to enhance their clarity and visibility.
