# Supplementary material for: Analysis of the antimicrobial activity of zinc oxide nanoparticles against drug-resistant bacteria and their applications in the disinfection process
Source: PLoS One. 2026 Feb 13;21(2):e0340470. doi: 10.1371/journal.pone.0340470 (PMC12904420; doi:10.1371/journal.pone.0340470)
Supplement: Attachment — Submitted filename: Response to reviewer 10-7-2025.docx [file pone.0340470.s006.docx]

**Response to reviewer**

**Reviewer #2:** Although authors have addressed maximum points raised however, I would recommend incorporating following points before the publications of the article.

**Recommendation: Minor Revision**

**Comment 1.** The authors should provide more information in the figure legends for non-experts. All legends should have enough description for a reader to understand the figure without having to refer back to the main text of the manuscript. For example, the necessary expansion (for abbreviations) should be given which are used in the present investigation.

**Response 1:** We greatly appreciate your comments , All figure legends in the article have been modified as requested. Thank you for your comments. Please read the updated legends for all figures in the revised manuscript file. Also please noticed that the entire figure 1 has been revised to simplify and clarify the experimental setup. The name of the ASB utilized in this study were indicated in Table 1. In addition, while modifying the legend for figure 5 highlighted the need for further clarification in the Materials and Methods section related to this figure. We have revised the text to ensure that even readers without prior experience can understand the outlined procedure and the results obtained.

**Comment 2 and 3.** Update the introduction section and cite this recent literature to attract a broad readership in the area i) Low-Friction Soft Robots for Targeted Bacterial Infection Treatment in Gastrointestinal Tract, ii) Pharmacokinetics effects of chuanxiong rhizoma on warfarin in pseudo germ-free rats, iii) Self-assembly multifunctional DNA tetrahedron for efficient elimination of antibiotic-resistant bacteria. While discussing medicinal properties of nanoparticles cite i) Advancing Aggregation-Induced Emission-Derived Biomaterials in Viral, Tuberculosis, and Fungal Infectious Diseases, ii) Dual recombinase polymerase amplification system combined with lateral flow immunoassay for simultaneous detection of Staphylococcus aureus and Vibrio parahaemolyticus

Response 2 and 3: Thank you for your comment, all the requested previous literature are now cited in the revised manuscript version. Please see page 32 paragraph 2.

*We would like to sincerely thank you for your thoughtful and constructive comments. We highly appreciate the time and effort dedicated to evaluating our manuscript and providing valuable feedback. We have carefully considered each point raised and have done our best to address all comments thoroughly and clearly. If we have misunderstood any of the suggestions or failed to fully meet any expectations, we remain open and committed to making any additional revisions necessary to improve the quality and clarity of our work.*
